# The Expanding Arsenal of Cytotoxic T Cells

**DOI:** 10.3389/fimmu.2022.883010

**Published:** 2022-04-20

**Authors:** Chiara Cassioli, Cosima T. Baldari

**Affiliations:** Department of Life Sciences, University of Siena, Siena, Italy

**Keywords:** lytic granule, SMAP, FasL, cytotoxic T cell, granzyme, perforin

## Abstract

Cytotoxic T cells (CTLs) are the main cellular mediators of the adaptive immune defenses against intracellular pathogens and malignant cells. Upon recognition of specific antigen on their cellular target, CTLs assemble an immunological synapse where they mobilise their killing machinery that is released into the synaptic cleft to orchestrate the demise of their cell target. The arsenal of CTLs is stored in lysosome-like organelles that undergo exocytosis in response to signals triggered by the T cell antigen receptor following antigen recognition. These organelles include lytic granules carrying a cargo of cytotoxic proteins packed on a proteoglycan scaffold, multivesicular bodies carrying the death receptor ligand FasL, and the recently discovered supramolecular attack particles that carry a core of cytotoxic proteins encased in a non-membranous glycoprotein shell. Here we will briefly review the main features of these killing entities and discuss their interrelationship and interplay in CTL-mediated killing.

## Introduction

Cytotoxic T lymphocytes (CTL) are the arm of the adaptive immune system specialised in killing virally infected or malignant cells. They are classically derived from CD8^+^ naive T cells that undergo a complex differentiation program following antigen recognition (1, 2), although CD4^+^ cells can also become cytotoxic effectors (3). CTLs trigger the apoptotic demise of their cell targets by exploiting a diversified arsenal of cytotoxic mediators stored in organelles, or “lytic granules” (LG) that are released on target cell recognition into the synaptic cleft, a space that forms at the highly organized interface of the CTL with its target (4).

Since the seminal discovery that LGs are secretory lysosomes carrying a cargo of proteases and the pore-forming protein perforin (Prf) that assists their delivery to the CTL target (5), the scenario has become significantly more complex. Other lysosome-related organelles (LRO) (6, 7) have been identified that contribute to the killing ability of CTLs, including extracellular vesicles (EV) carrying the apoptosis-inducing factors Fas ligand (FasL) and APO2 ligand (APO2L)/TRAIL generated in multivesicular bodies (MVB) (8–10) and, more recently, the supramolecular attack particles (SMAPs) originating from a new, as yet only partly characterised LRO (11, 12). Here we will briefly review the three known classes of LRO exploited by CTLs for killing and discuss their specific role in this process to confer CTLs the ability not only to efficiency eliminate individual target cells, but also to make them powerful serial killers.

## The Arsenal of CTLs

### Lytic Granules

LGs were initially characterised as killing entities of 300-1100 nm consisting of an electron-dense core often surrounded by 30-70 nm vesicles enclosed by the delimiting outer membrane (5). LG fractionation allowed for the identification of their cytotoxic contents, which consist of a battery of serine proteases with different substrate specificity, the granzymes (Gzm) (13), and Prf, a protein with structural and functional homology to bacterial pore-forming toxins (14), packed together on a scaffold of the proteoglycan serglycin (Srgn) (15) in the dense core. Additionally, LGs contain the processed, 9 kDa isoform of granulysin, a saposin-like membrane-disrupting protein (16). Several pieces of evidence witness to the lysosomal origin of LGs, including the low pH and the presence of lysosomal hydrolases (e.g. cathepsin D) and of lysosomal membrane glycoproteins (e.g. LAMP-1) (5, 17). The multivesicular cortex of LGs is also enriched in the cation-independent mannose-6-phosphate receptor (CI-MPR), which transports the acid hydrolases to lysosomes (18). The multivesicular structure of LGs is generated through the invagination of the membrane of early endosomes (EE) (19), the sorting hub for proteins endocytosed at the cell surface, which accounts for the identification of plasma membrane-associated proteins such as T cell receptors (TCRs) and integrins in the multivesicular cortex (17) and highlights the endolysosomal origin of these organelles.

**Figure 1:**
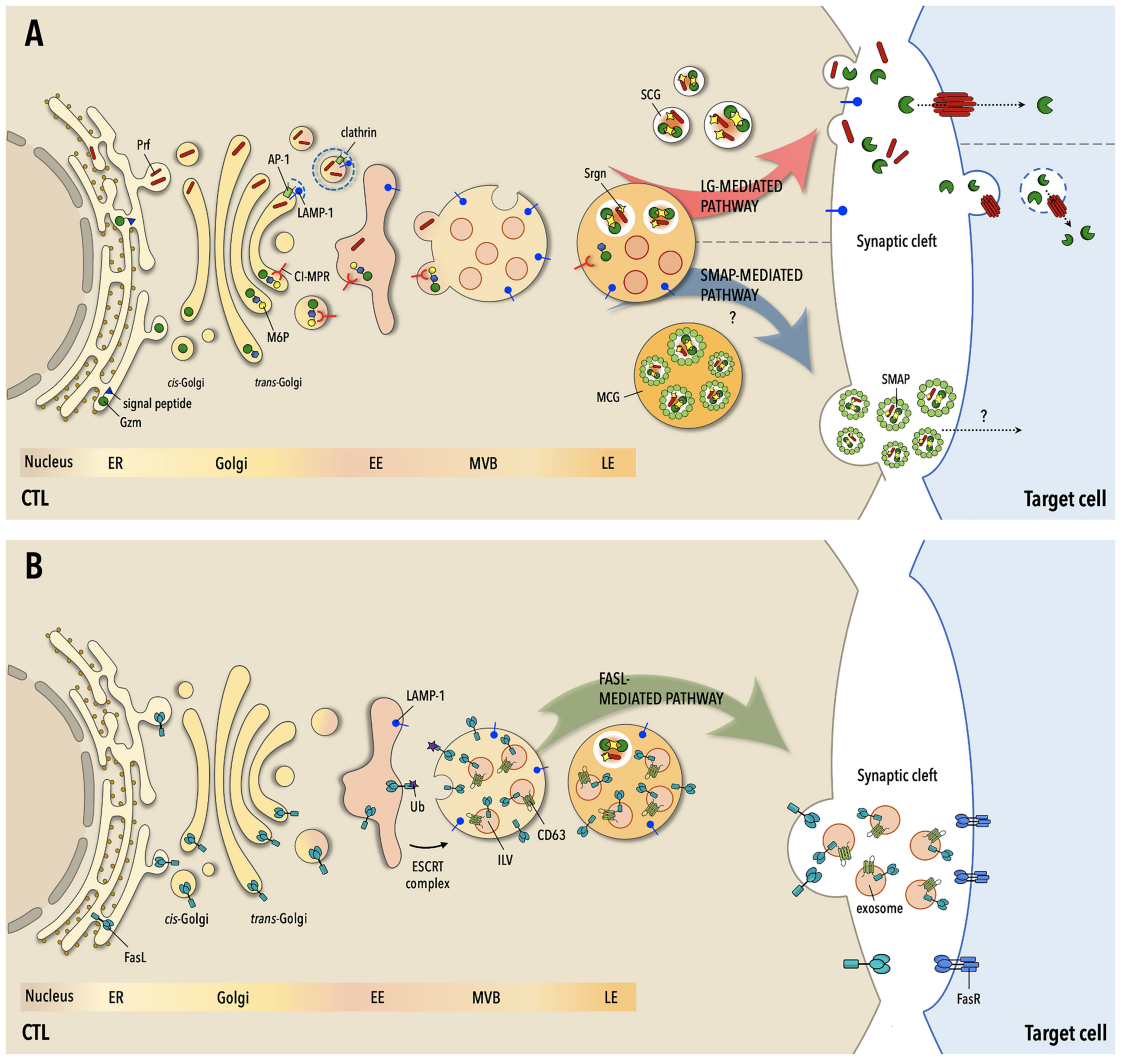
The three pathways to target cell killing by CTLs. **(A)** Following routing to the secretory pathway at the endoplamic reticulum (ER), the components of lytic granules (LG) -granzymes (Gzm), perforin (Prf) and serglycin (Srgn)- are sorted at the Golgi apparatus for transport to early endosomes (EE), wherefrom they transit through multivesicular bodies (MVB) and late endosomes (LE) to mature LGs. Gzms are tagged for transport by the cation-independent mannose-6P receptor (CI-MPR) through N-glysosylation and the addition of a M-6P moiety, while Prf is sorted through clathrin-dependent and -independent pathways. At LEs Gzms and Prf become activated but remain in an inactive state until their release and eventually localize in two types of mature LGs, single-core granules (SCG) or multiple-core granules (MCG), accumulating as multimolecular complexes held together by Srgn. Upon formation of the immunological synapse (IS) with the cognate cell target SCGs are mobilized to the cell-cell contact and fuse with the IS membrane, releasing soluble Gzm-Prf complexes that are taken up by the target cell through the pore-forming activity of Prf. In MSGs Gzm-Prf-Srgn complexes are encased in a glycoprotein shell enriched in thrombospondin-1 to form the SMAPs. Following CTL activation, MCGs undergo fusion with the IS membrane with a delayed kinetics compared to SCGs and release their cargo of SMAPs, which are taken up by the cell target through an as yet unidentified mechanism. **(B)** FasL transits through the ER, Golgi apparatus and EEs to MVBs, where it becomes associated both to the limiting membrane and to intraluminal vesicles that mature into EVs. FasL may also be partly segregated to Gzm- and Prf-containing LGs. On encounter of their cognate cell target CTLs mobilize MVBs to the IS, releasing FasL both at the synaptic membrane and into the synaptic cleft as FasL-containing EVs. Both plasma membrane-associated and EV-associated FasL can interact with Fas on the target cell membrane, triggering the Fas- and caspase-dependent death pathway. Fas-dependent killing is delayed compared to Gzm/Pfr-dependent killing and is essential for the serial killing activity of CTLs.

The pathways that regulate LG biogenesis downstream of expression of their components have been in part elucidated (**Figure 1**). Granzymes, of which the best characterized is GzmB, enter the endoplasmic reticulum (ER) as inactive precursors that are sorted at the trans-Golgi network (TGN) by the CI-MPR (20) following N-glycosylation and acquisition of a mannose-6-phosphate moiety, and bud off in endocytic carriers in a process involving the endosomal sorting complex required for transport (ESCRT) machinery (21). Gzms are first transported to EEs, wherefrom they are sorted into MVBs and directed to late endosomes (LE) (19). At the low local pH they complete their proteolytic maturation but are maintained inactive both by the low pH and through their sequestration by Srgn in the dense core (13). By contrast, the pathway that regulates Prf transport to maturing LGs is as yet poorly characterized. A molecular determinant within its C-terminus and N-linked glycosylation (22), as well as LAMP-1 interaction with the AP-1 sorting complex (23), are required for its efficient export from the ER and LG localisation. Multiple levels of control ensure that Prf is kept inactive until release upon CTL activation. To polymerise and form pores on biological membranes Prf requires Ca^2+^ binding, which is prevented by the low pH of LGs. Additionally, Prf is kept in a monomeric form by Srgn binding (24). As opposed to the lytic components of the dense core, integral LG membrane proteins such as LAMP-1 or LAMP-2 are sorted at the TGN through canonical tyrosine-based or di-leucine-based motifs that are recognized by AP-1 (25).

TCR engagement on CTLs triggers centrosome polarisation towards the contact with the target cell and its close apposition to the synaptic membrane in a process tightly regulated by the actin and tubulin cytoskeletons, which sets the stage for the dynein- and AP-3-dependent transport of LGs to the immunological synapse (IS) center (26, 27), as well as by TCR signal strength (28). Interestingly, rapid LG secretion on target cell encounter can occur in the absence of centrosome polarisation, which is instead essential for the establishment of stable, stimulatory synapses (29), suggesting an alternative mechanism for LG mobilisation to the target cell contact.

Gzm- and Prf-enriched LGs acquire the ability to dock to the plasma membrane and deliver their cytotoxic cargo into the synaptic cleft through two sequential maturation steps that have been extensively characterized following the identification of gene mutations responsible for primary immunodeficiency disorders associated with defective CTL function (30). LG docking to the synaptic membrane requires the acquisition of two key trafficking regulators: the Rab GTPase Rab27 and its effectors synaptotagmin-like proteins SLP1/2, and the adaptor Munc13-4 (31–37). According to one model, this involves fusion of maturing LGs with an intermediate exocytic vesicle generated through the Munc13-4-dependent fusion of recycling endosomes (RE), which carry Munc13-4, with LEs, which carry Rab27a (37). The resulting docking-competent LGs exploit the phospholipid-binding ability of SLP1/2 to interact with the synaptic membrane. Docked LGs become fusogenic through a priming step, which also depends on Munc13-4 (37). This involves the formation of a complex between the vesicle-soluble NSF attachment protein receptor (v-SNARE) VAMP7 at the LG membrane and the target-soluble NSF attachment protein receptor (t-SNARE) syntaxin 11 and its partners SNAP23 and Munc18-2 at the plasma membrane (38–40). More recent data indicate that, rather than fusing before delivery to the IS, REs carrying Rab27 and Munc13-4 and LGs polarise to the IS independently and undergo sequential fusion (41). Accordingly, syntaxin 11 has also been shown to be transported to the IS by REs and released with the assistance of the v-SNARE VAMP8, thereby marking the location for LG docking (42).

Once released into the synaptic cleft, Prf and Gzms cooperate to promote the apoptotic demise of the CTL target. Although the key role for Prf in the delivery of Gzms to the cognate target has been well established, different mechanisms have been proposed, all involving the pore-forming activity of Prf, the polymerisation of which is enabled by its dissociation from Srgn at the higher pH of the synaptic cleft and Ca^2+^ binding: i) delivery of Gzms to the cytosol of the target cell through Prf pores, either directly at the synaptic membrane (43) or following their co-internalisation in endosomes (44, 45); or ii) internalisation of Gzms as a membrane repair response triggered by Prf-induced membrane damage (46). In the cytosol Gzms induce target cell apoptosis by activating caspase-dependent and caspase-independent pathways (13). Granulysin also contributes to the cytotoxic activity of LGs by interacting with the target cell membrane through its positive charges and inducing the influx of Ca^2+^, which leads to mitochondrial damage and caspase-3 activation (16).

### FasL Granules

In addition to their LG-dependent cytotoxic activity, CTLs exploit the Fas pathway to kill target cells (47). CTLs express FasL, a type II transmembrane protein that is upregulated at the cell surface in response to target cell recognition. Similar to Gzms and Prf, pre-formed FasL is stored in LROs that had been proposed to correspond to LGs, based on its co-localisation with LG markers and the observation that FasL delivery to the cell surface was dependent on degranulation (48). More recent findings indicate that FasL, while indeed stored in LROs, is associated with granules that appear distinct from canonical, dense-core LGs (49). Proteomic analyses identified two subpopulations of cytotoxic granules of different size, of which the larger (300-700 nm) is enriched in FasL and lysosome/MVB markers, and the smaller (<300 nm) in Gzms and Prf (50). Additionally, release of pre-stored FasL was found to have a lower TCR signal threshold than LG release, to be microtubule- and extracellular Ca^2+^-independent, and under certain stimulation conditions to occur in the absence of degranulation (49, 51, 52). The association of intracellular FasL with two populations of LGs, as documented in NK cells (53), may account for these discrepancies.

FasL is targeted to MVB, where it localises together with APO2L/TRAIL both at the limiting membrane and in CD63^+^ intraluminal vesicles (ILV) that are released into the synaptic cleft as EVs (8, 9, 48, 54). Sorting of FasL into MVBs is regulated by phosphorylation by Src kinases that interact with its proline-rich domain and by mono-ubiquitylation (10, 55) which allows for its routing to the ESCRT pathway (21). In this pathway proteins destined for LEs and lysosomes are sorted into ILVs at EEs through recruitment of ESCRT-0, which binds phosphatidylinositol 3-phosphate (PI3P) on the endosomal membrane and mono-ubiquitylated proteins, followed by sequential binding of ESCRT-I and ESCRT-II to nucleate ESCRT-III filaments. These drive the process of membrane deformation that is essential for invagination and pinching off of ILVs (21).

Upon target cell recognition, MVBs polarise towards the centrosome and undergo fusion with the synaptic membrane in a process that in T cells is regulated by diacylglycerol kinase α and the tetraspanin MAL (56, 57). Depending on its localisation within the MVB, FasL is either redistributed to the plasma membrane or released in association with EVs. Using supported lipid bilayers (SLB) to allow for tight control of target membrane composition, Balint and colleagues showed that FasL^+^ puncta were present in the synaptic cleft only when Fas was included in the SLB (11). While this indicates that FasL is associated to EVs, how their delivery is linked to the availability of Fas at the target membrane is not clear. A possible mechanism is suggested by the requirement of CD40 in the SLB for the synaptic release of CD40L^+^ vesicles from helper T cells, which is triggered by co-clustering of CD40L-CD40 with TCR-peptide major histocompatibility complex (pMHC) (58). Whether plasma membrane- or EV-associated, FasL can engage Fas on the target cell, triggering the assembly of a signaling complex that leads to activation of the apoptotic cascade (47).

### SMAPs

The recent discovery of SMAPs (11) has unveiled a third, unconventional mechanism of CTL-mediated cytotoxicity, which is shared by NK cells (59). Balint and colleagues used SLBs functionalised with anti-CD3 mAbs and ICAM-1 to activate CTLs and observed that, after CTL removal, glycoprotein complexes -the SMAPs- were left behind. A proteomic analysis of SMAPs revealed an unexpected composition featuring a lack of membrane proteins and an enrichment in canonical LG effectors (Prf, Gzms, Srgn), as well as glycoproteins, of which thrombospondin-1 (TSP-1) and galectin-1 were prominent components. Super-resolution imaging and structural analyses showed that SMAPs are highly stable ∽120 nm particles, with the lytic effectors concentrated in a core surrounded by a glycoprotein shell. Consistent with their lytic cargo, purified SMAPs have the ability to kill cells autonomously (11).

Within CTLs SMAPs are stored in multicore granules (11). A recent report by the Rettig lab has shed light on the identity of this LG population. Using a mouse knock-in for fluorescently tagged Synaptobrevin2 (Syb2), the murine homologue of VAMP7 that marks fusion-competent LGs, Chang and colleagues (12) purified and characterized mature LGs. They identified two distinct Syb2^+^ populations that were confirmed to be LGs based on co-fractionation and co-localization with GzmB: a homogeneous population of smaller granules with a single dense core (SCG), and a heterogenous population of larger granules with multiple dense cores (MCG). Proteomic analysis revealed a remarkably different composition of SCGs and MCGs, with SCGs enriched in lysosomal proteins and MCGs in endosomal trafficking regulators. TSP-1 was found to selectively associate with MCGs and to be released in particles with a similar core-shell structure as the human CTL-derived SMAPs. SCGs and MCGs release involved distinct fusion events, suggesting that the two classes of LG mature and undergo exocytosis through independent pathways (12).

## How Many Pathways to CTL-Mediated Killing?

While revealing a new weapon in the CTL killing arsenal, the discovery of SMAPs and of MCGs as their putative intracellular storage compartment has added further complexity to the current view of the mechanisms of CTL-mediated killing, from the biogenesis of the LROs that store their killing effectors, to the transport, release and uptake of each class of LRO at the IS formed with their cognate cell target, to their interplay in cytotoxicity.

A first open question is whether different classes of mature Gzm^+^Prf^+^ LGs co-exist in CTLs. Until recently the most mature LGs were considered those with a single electron-dense core, which could correspond to the SCGs described by Chang et al. (12), while larger LGs with a single dense core surrounded by ILVs were considered a more immature stage downstream of MVBs in the LG maturation pathway (30). The discovery of MCGs as a distinct class of mature LGs that are Gzm^+^Prf^+^TSP-1^+^ (12) has challenged this view, suggesting branching of the LG biogenesis pathway downstream of the accumulation of the cytotoxic effectors, followed by SMAP biogenesis selectively in the MCGs. While the existence of SCGs and MCGs remains to be demonstrated in human CTLs, it is supported by the observation that SMAPs accumulate in MCG-like organelles in these cells (11). The different timing of exocytosis of canonical LGs and SMAPs (11) reinforces the notion that they are independent killing entities exploiting distinct exocytic pathways for release in the synaptic cleft.

Whether FasL, the other main cytotoxic effector of CTLs, is stored in the same LROs where Prf/Gzms/Srgn, are stored is a related, as yet open question. FasL has been initially reported as co-localising with Gzm^+^Prf^+^ granules, with a preferential association with ILVs that surround the dense core enriched in Prf/Gzms/Srgn complexes (48) and are released as FasL^+^ EVs when LGs fuse by the synaptic membrane (8). This view has been challenged by the finding that in mouse CTLs the intracellular FasL pool is localised in vesicles distinct from Gzm^+^Prf^+^ LGs that are mobilised to the cell surface independently of degranulation (49). This notion is supported by the identification in human CTLs of two classes of LGs with a different protein composition, of which one enriched in Prf/Gzms and the other in FasL (50). To further confound the picture, FasL was not found in either SCGs or MCGs in mouse CTLs, at least by proteomics (12). In-depth investigation of the pathways regulating the biogenesis and maturation of these different LROs is essential to clarify their interrelationship and interplay in CTL cytotoxicity. In this context, it will be important to address how signal 3 (IL-12, IFN-α), which has been shown to be essential for the acquisition of cytolytic function by CD8^+^ T cells (60, 61), impacts on the biogenesis of each of these killing entities.

A second question is whether the three cytotoxicity pathways, of which two mediated by the same effectors -Gzms and Prf- delivered in either soluble form or as SMAPs, and the third by plasma membrane- or EV-associated FasL activating the death pathway, have unique or redundant roles in killing. The fact that the demise of cancer cells, such as melanoma cells, is delayed and less efficient compared to normal cells (62) also due to the deplyoment of counterattack strategies such as secretion of FasL^+^ and APO2L/TRAIL^+^ EVs (63), suggests a requirement for all three mechanisms for efficient killing. Based on the time required for Gzm/Prf- versus FasL/Fas-dependent killing (64, 65), it has been proposed that the initial individual killing events are triggered by the Gzm/Prf pathway, while the FasL/Fas pathway is mainly responsible for the subsequent multiple killing events. Using reporters that allow to discriminate between Gzm-mediated and FasL-mediated killing in NK cells, Prager and colleagues have shown that these cells switch from GzmB to caspase-8 (a marker of the apoptosis pathway triggered by FasL) during serial killing, and that this ability was impaired in FasL-deficient cells (66). This tight coordination of the two killing pathways is likely to be shared by CTLs, although the requirement for *de novo* synthesis of GzmB and Prf for serial killing, recently shown to involve mitochondrial control of their mRNA translation (67), indicates that this model may have to be reassessed. How SMAPs enter the picture remains to be understood. SMAPs are autonomous, long-lived killing entities (11), similar to FasL^+^ EVs. Hence they may act as slow-release devise for Gzms and Prf after their soluble counterparts have been rapidly taken up by the cognate cell target at the synaptic cleft. While this suggests that SMAPs may contribute both to the effective elimination of the initial CTL target and to the process of serial killing, the requirement for SMAPs in CTL-mediated killing and the underlying mechanisms need to be elucidated to answer this question.

## Conclusions and Perspectives

CAR T cell-based immunotherapy has emerged as a powerful strategy to mobilise the killing potential of CTLs to specifically target tumor cells. However, despite spectacular results in the treatment of haematological malignancies, CAR T cell therapy poses major hurdles due to adverse effects that include excessive, life-threatening production of proinflammatory cytokines, limited ability of CAR T cell penetration into solid tumors and disabling by suppressive factors produced by the tumor microenvironment (68). A promising alternative to CAR T cell therapy is based on the finding that, upon activation, T cells release MVB-derived EVs that display TCR complexes, integrins and FasL on their limiting membrane (9, 17, 69) and are enriched in cytotoxic components of LGs (70). Pre-clinical studies showed that these EVs can kill tumor cells carrying cognate pMHC (71, 72). This has led to the idea of translating the advantages of CAR T cell therapy to a CAR T cell-derived EV-based therapy, which maintains the specific anti-tumor activity of the cell of origin while bypassing the CAR T cell-related adverse effects. The potential of this approach is underscored by the flurry of studies in a variety of disease contexts and by the progress in the development of modified or synthetic EVs that incorporate selected cargo. SMAPs, with their cargo of toxic molecules and their long half-life, could represent an attractive new CTL-free immunotherapeutic for cancer treatment if provided with the ability to recognize specific tumor antigen by engineering the glycoprotein shell. Dissecting the pathways that regulate the biogenesis of all the cytotoxic particles produced by CTLs and NK cells will help designing new, robust and safe anticancer therapies.

## Author Contributions

CB and CC wrote the paper. CC prepared the figure. All authors contributed to the article and approved the submitted version.

## Funding

The support of AIRC (grant IG-20148) and EU (ERC-2021-SyG 951329) is gratefully acknowledged.

## Conflict of Interest

The authors declare that the research was conducted in the absence of any commercial or financial relationships that could be construed as a potential conflict of interest.

## Publisher’s Note

All claims expressed in this article are solely those of the authors and do not necessarily represent those of their affiliated organizations, or those of the publisher, the editors and the reviewers. Any product that may be evaluated in this article, or claim that may be made by its manufacturer, is not guaranteed or endorsed by the publisher.
